# Relative contributions of non-essential Sec pathway components and cell envelope-associated proteases to high-level enzyme secretion by *Bacillus subtilis*

**DOI:** 10.1186/s12934-020-01315-2

**Published:** 2020-02-28

**Authors:** Jolanda Neef, Cristina Bongiorni, Brian Schmidt, Vivianne J. Goosens, Jan Maarten van Dijl

**Affiliations:** 1 Department of Medical Microbiology, University of Groningen, University Medical Center Groningen, Hanzeplein 1, P.O. Box 30001, 9700 RB Groningen, The Netherlands; 2 DuPont Industrial Biosciences, 925 Page Mill Road, Palo Alto, CA 94304 USA

**Keywords:** *Bacillus subtilis*, DnaK, HtrA, HtrB, Protein production, Protein translocation, RasP, SecDF, SecG, Secretion stress

## Abstract

**Background:**

*Bacillus subtilis* is an important industrial workhorse applied in the production of many different commercially relevant proteins, especially enzymes. Virtually all of these proteins are secreted via the general secretion (Sec) pathway. Studies from different laboratories have demonstrated essential or non-essential contributions of various Sec machinery components to protein secretion in *B. subtilis*. However, a systematic comparison of the impact of each individual Sec machinery component under conditions of high-level protein secretion was so far missing.

**Results:**

In the present study, we have compared the contributions of non-essential Sec pathway components and cell envelope-associated proteases on the secretion efficiency of three proteins expressed at high level. This concerned the α-amylases AmyE from *B. subtilis* and AmyL from *Bacillus licheniformis*, and the serine protease BPN’ from *Bacillus amyloliquefaciens*. We compared the secretion capacity of mutant strains in shake flask cultures, and the respective secretion kinetics by pulse-chase labeling experiments. The results show that *secDF*, *secG* or *rasP* mutations severely affect AmyE, AmyL and BPN’ secretion, but the actual effect size depends on the investigated protein. Additionally, the chaperone DnaK is important for BPN’ secretion, while AmyE or AmyL secretion are not affected by a *dnaK* deletion. Further, we assessed the induction of secretion stress responses in mutant strains by examining AmyE- and AmyL-dependent induction of the quality control proteases HtrA and HtrB. Interestingly, the deletion of certain *sip* genes revealed a strong differential impact of particular signal peptidases on the magnitude of the secretion stress response.

**Conclusions:**

The results of the present study highlight the importance of SecDF, SecG and RasP for protein secretion and reveal unexpected differences in the induction of the secretion stress response in different mutant strains.

## Background

The Gram-positive bacterium *Bacillus subtilis* and related bacilli are well known producers of secreted enzymes. These bacteria have excellent fermentation properties, and they deliver enzyme yields of over 25 g per liter culture in industrially optimized processes [1]. The secrets underlying these commercially significant secreted enzyme yields are hidden in a highly efficient protein secretion machinery and the relatively simple cell envelope structure that characterizes Gram-positive bacilli.

The *Bacillus* cell envelope is composed of a thick cell wall, consisting of peptidoglycan and other polymers, such as (lipo-) teichoic acids. Due to its porous structure, the cell envelope allows the diffusion of proteins that are translocated across the cytoplasmic membrane into the fermentation broth [2]. Additionally, the negative charge of cell wall polymers, especially the (lipo-) teichoic acids, contributes to protein secretion by retaining cations that facilitate the post-translocational folding of secretory proteins [2–4]. Importantly, due to the absence of an outer membrane, as present in Gram-negative bacteria, *Bacillus* products are endotoxin-free. Accordingly, many of these products, especially amylases and proteases, have been granted the Generally Regarded as Safe (GRAS) status by the United States Food and Drug Administration (FDA) [5–7].

In *Bacillus* species, protein secretion is predominantly facilitated by the general secretion (Sec) pathway, which comprises components that convert energy in the form of ATP and the transmembrane proton-motive force into a mechanical force that drives proteins through a membrane-embedded channel. The Sec pathway can effectively handle many different secretory proteins and, since the downstream processing of secreted proteins from the fermentation broth is fairly straightforward, this pathway is extensively exploited in the biotechnology industry [5, 8].

The subsequent stages in Sec-dependent protein secretion ‘from the ribosome to the growth medium’ require different secretion machinery components many of which are essential for cell growth and viability. These components include the signal recognition particle (especially required in membrane protein biogenesis), the core components of the Sec translocase that facilitates the actual membrane passage of secretory proteins in an unfolded state, and the post-translocational protein folding catalyst PrsA [9–15]. On the other hand, the Sec pathway also includes various non-essential components that modulate the efficiency of protein export. These include general chaperones that modulate protein folding in the cytoplasm like DnaK [16, 17], translocase components like SecG and SecDF [18–20], and signal peptidases (SipS-W) that liberate Sec-translocated proteins from the membrane [21–23]. Several factors are not directly involved in the protein export process but are, nonetheless, needed for its optimal performance. These include potential signal peptide peptidases, like TepA, SppA and RasP, [24–26], and quality control proteases like HtrA, HtrB and WprA [27–32]. TepA, SppA and RasP have been implicated in degradation of cleaved signal peptides, and in keeping the membrane clear from mistranslocated or misassembled proteins [24–26]. HtrA, HtrB and WprA remove aggregated or malfolded proteins from the membrane-cell wall interface or the cell wall and they may contribute to folding of translocated proteins as well [27–32].

Accumulation of malfolded proteins due to high-level protein production is sensed by the membrane embedded two-component regulatory system CssRS [28, 33]. Activation of the sensor kinase CssS by high-level secretion of amylases or by heat stress leads to phosphorylation of the CssR response regulator and subsequent induction of the membrane-attached quality control proteases HtrA and HtrB, which also have a chaperone activity [28–30]. N-terminally cleaved forms of HtrA and HtrB can also be encountered in the growth medium, but they are subject to degradation by secreted proteases of *B. subtilis* [34–36]. Of note, *htrA* and *htrB* are CssRS-dependently cross-regulated, which means that one is upregulated when the other is deleted [37, 38]. This indicates that basal levels of HtrA and HtrB production are needed to avoid secretion stress. Intriguingly, the protease WprA serves an important function at the membrane-cell wall interface controlling not only the levels of secretory proteins but also of the protein folding catalyst PrsA [37, 39, 40].

In previous studies as referenced above, the roles of individual Sec machinery components and cell envelope-associated proteases have been analyzed in great detail. However, this was often done with different secretory reporter proteins in different genetic backgrounds, and a systematic comparison of the impact of each individual Sec machinery component under conditions of high-level protein secretion was so far missing. Such a systematic comparison is challenging for essential secretion machinery components due to the high risk of indirect effects upon their depletion. However, this kind of analysis is perfectly feasible for the non-essential secretion machinery components. In the present study, we have therefore compared the contributions of non-essential Sec pathway components and cell envelope-associated proteases of *B. subtilis* on the secretion efficiency of three proteins expressed at high levels. Specifically, this concerned the α-amylases AmyE from *B. subtilis* and AmyL from *Bacillus licheniformis*, and the serine protease BPN’ from *Bacillus amyloliquefaciens*, which are representative for a large group of commercially relevant industrial enzymes. Briefly, the results show that deficiencies of SecDF, SecG or RasP have the strongest negative impact on the secretion of these reporter enzymes. In addition, we show that a DnaK deficiency has a negative impact on the rate of BPN’ secretion.

## Results

### Base-line secretion levels of the AmyE, AmyL and BPN’ reporter proteins

**Fig. 1:**
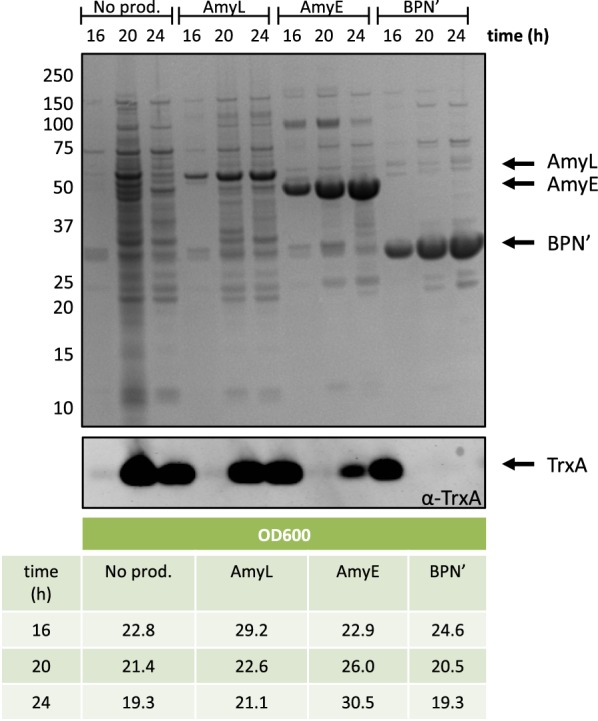
Secretion of AmyL, AmyE and BPN’ upon 16, 20 or 24 h of growth. Cells were separated from the growth medium by centrifugation after 16, 20 or 24 h of growth in MBU medium at 37 °C. Subsequently, proteins in the growth medium fractions were precipitated with TCA, separated by LDS-PAGE, and visualized with SimplyBlue SafeStain (upper panel). Prior to TCA precipitation and gel loading, the samples were corrected for the OD_600_ of the respective cultures as listed in the bottom panel. To assess the extent of cell lysis during culturing, the extracellular levels of the cytoplasmic marker protein TrxA were assessed by Western blotting with specific antibodies (middle panel). Molecular weights of marker proteins are indicated (in kDa) on the left side of the gel segment

The present study was aimed at a systematic examination of the impact of non-essential secretion machinery components of *B. subtilis* on the secretion of two α-amylases, namely AmyE and AmyL and the serine-protease BPN’. To exclude differential effects on the secretion of these three reporter proteins due to the usage of different expression or secretion signals, the *amyE*, *amyL* and *bpn*’ genes were inserted in the chromosomal *aprE* locus, transcribed from the *aprE* promoter, and provided with the *aprE* signal sequence that directs Sec-dependent secretion [26]. The use of the strong DegU-controlled *aprE* promoter has the additional advantage that it is highly activated in a so-called *degU*32(Hy) mutant background, where DegU is constitutively phosphorylated [41]. Accordingly, strains containing these expression modules and the *degU*32(Hy) mutation can secrete high levels of AmyE, AmyL or BPN’ into the growth medium [26]. This is exemplified in Fig. 1 (upper panel), showing a SimplyBlue-stained gel with AmyE, AmyL or BPN’ produced by the *degU*32(Hy) mutant parental strain used in this study. For this particular experiment, the bacteria were grown in MBU medium under fermentation-mimicking conditions, and samples for lithium dodecyl sulfate (LDS) PAGE were withdrawn after 16, 20 or 24 h of growth. Of note, at 20 or 24 h of growth, the highest extracellular levels of AmyE, AmyL and BPN’ were observed, but at these time points the bacteria were prone to significant cell lysis as was evidenced by Western blotting for the cytoplasmic marker protein TrxA (Fig. 1, middle panel). Only in the case of BPN’ secretion no extracellular TrxA was observed, but this is probably due to the degradation of this marker protein by the highly active BPN’ protease. To minimize unwanted side effects of cell lysis, in all further experiments the bacteria were grown for about 16 to 17 h at which time point the optical densities of the cultures at 600 nm (OD_600_) were comparable but not identical (Fig. 1, lower panel).

### SecDF, SecG and RasP are of major importance for extracellular protein yields

To systematically compare the effects of non-essential secretion machinery components, we constructed a series of isogenic strains lacking the genes for the chaperone DnaK, the translocase subunits SecDF or SecG, or the signal peptidases SipS, SipT, SipU, SipV or SipW. In addition, we constructed isogenic strains lacking the genes for the cell envelope-associated proteases SppA, TepA, PrsW, WprA, YqeZ, HtrA or HtrB, which have established or potential roles in membrane or secretory protein quality control [35]. A previously characterized strain lacking the *rasP* gene was included to serve as a control in which the secretion of AmyE, AmyL and BPN’ is severely affected [26].

**Fig. 2:**
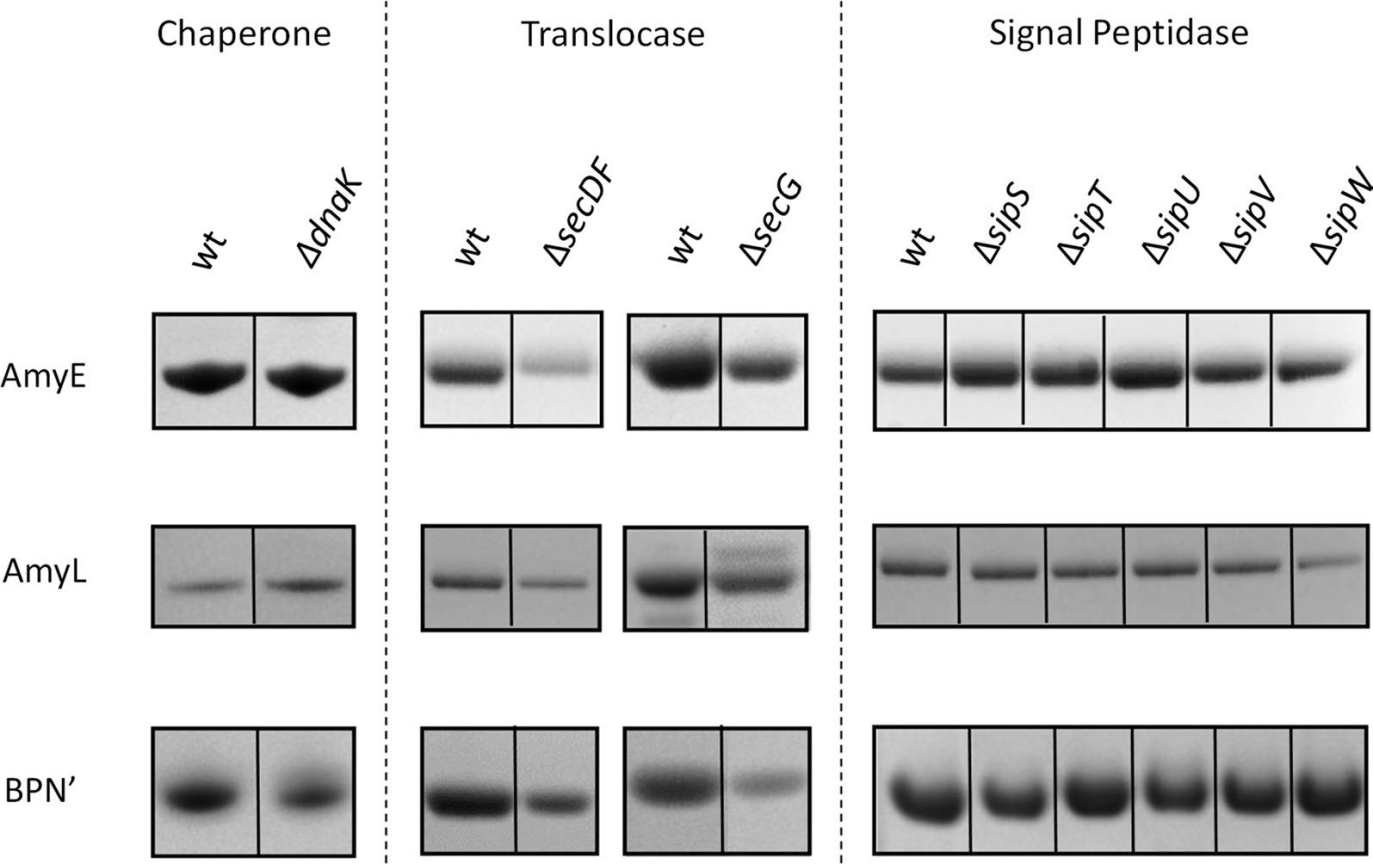
Secretion of AmyE, AmyL or BPN’ by strains lacking individual non-essential secretion machinery components. AmyE-, AmyL- or BPN’-producing strains lacking the *dnaK*, *secDF*, *secG*, *sipS*, *sipT*, *sipU*, *sipV* or *sipW* genes, as well as the respective wild-type (wt) control, were grown for 16 h in MBU medium at 37 °C. Next, cells and growth media were separated by centrifugation and proteins in the growth medium fractions were analyzed by LDS-PAGE and SimplyBlue SafeStaining as described for Fig. 1

**Fig. 3:**
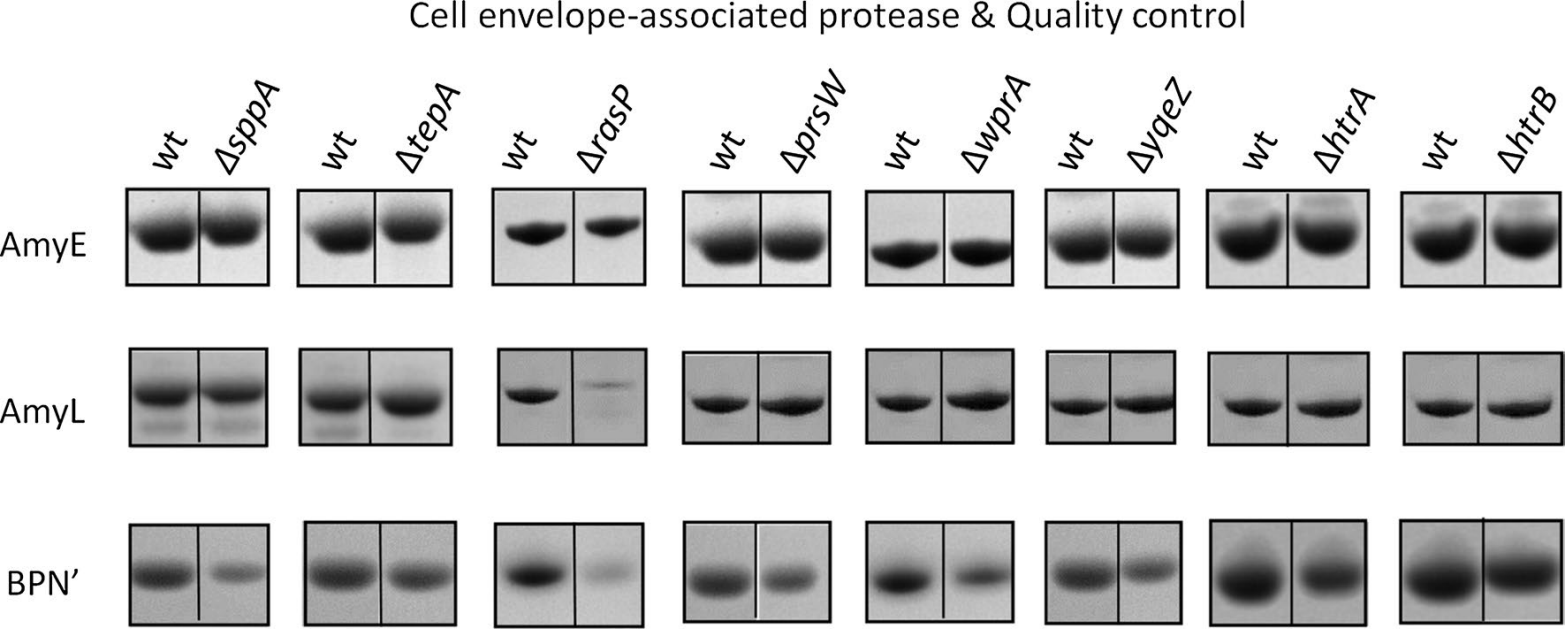
Secretion of AmyE, AmyL or BPN’ by strains lacking individual cell envelope-associated proteases. AmyE-, AmyL- or BPN’-producing strains lacking the *sppA*, *tepA*, *rasP*, *prsW*, *wprA*, *yqeZ*, *htrA* or *htrB* genes, as well as the respective wild-type (wt) control, were grown for 16 h in MBU medium at 37 °C. Next, cells and growth media were separated by centrifugation, and proteins in the growth medium fractions were analyzed by LDS-PAGE and SimplyBlue SafeStaining as described for Fig. 1. *, the effects of a *rasP* deletion were previously described [26]

As shown in Figs. 2 and 3, all strains lacking non-essential secretion machinery components or cell envelope-associated proteases did secrete AmyE, AmyL and BPN’. However, several of the investigated mutations impacted on the amounts of secreted protein as detectable by LDS-PAGE. This was especially evident for strains lacking *secDF*, where all three reporter proteins were secreted to severely reduced levels, consistent with previous observations for the amylase AmyQ [19]. Interestingly, in contrast to the finding by Bolhuis et al. that secretion of the neutral protease NprE was not affected by the *secDF* mutation [19], our present studies show that BPN’ secretion is reduced by this mutation.

In the case of a *secG* mutation, the yields of BPN’ and, to a somewhat lesser extent, AmyE and AmyL were reduced (Fig. 2), which is consistent with the previous finding by van Wely et al. that β-lactamase secretion was reduced in a *secG* mutant [20]. Further, as previously shown [26], the *rasP* mutation had drastic effects on the yields of secreted AmyL and BPN’, but to lesser extent on the yield of AmyE (Fig. 3).

For other investigated mutant strains, variations in the extracellular protein yields were detectable, but these were relatively mild compared to the effects observed for the *secDF*, *secG* and *rasP* mutations (Figs. 2 and 3). For instance, mutations in *sip* genes did influence AmyE and AmyL secretion to some extent (Fig. 2), consistent with previous findings reported for secretion of the *B. amyloliquefaciens* α-amylase AmyQ in *B. subtilis* [21, 23, 42]. Of note, the secretion of BPN’ was apparently affected by the *sppA* mutation, but this effect was variable in different experiments. Further, the *sppA* and *tepA* mutations did not affect AmyE or AmyL secretion, which is different from what was previously reported for AmyQ [24]. This shows that, apparently, SppA and TepA are not necessary for the efficient secretion of AmyE and AmyL, and it is in line with the observation that SppA may be more important for the protection against peptides with antimicrobial activity, especially lantibiotics [43].

Based on these observations, we conclude that SecDF, SecG and RasP are key non-essential determinants for extracellular protein production in *B. subtilis*. Importantly, however, the extent of the impact of SecDF, SecG or RasP varies substantially for different secretory proteins as exemplified here with AmyE, AmyL and BPN’.

### Reduced rates of protein export in *secDF*, *secG* and *dnaK* mutant cells

**Fig. 4:**
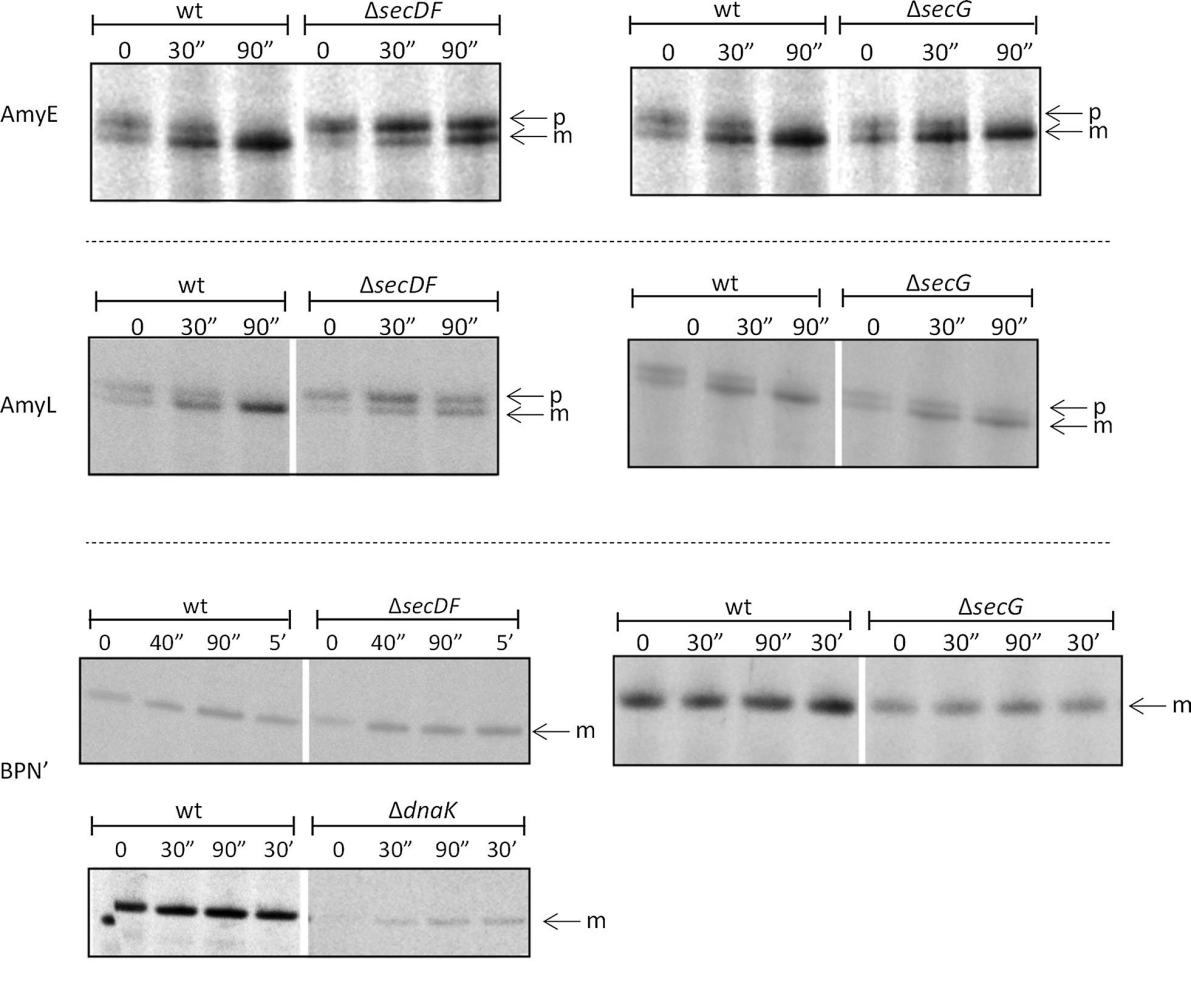
Kinetics of AmyE and AmyL precursor processing, and BPN’ secretion in *secDF*, *secG* or *dnaK* mutant strains. Processing of AmyE or AmyL precursors (p) to the respective mature forms (m) was analyzed by pulse-chase labeling. Cells grown in MBU medium at 37 °C were labeled with [^35^S]-methionine for 30 s prior to chase with excess non-radioactive methionine. Samples were withdrawn at the indicated time points after the chase and mixed with ice-cold TCA. Subsequently, (pre-)AmyE or (pre-)AmyL were immunoprecipitated with specific antibodies against AmyE or AmyL, separated by LDS-PAGE, and visualized by autoradiography. The secretion of BPN’ was also analyzed by pulse-chase labeling of cells grown in MBU at 37 °C with [^35^S]-methionine for 30 s prior to chase with excess non-radioactive methionine. However, in this case, samples withdrawn at the indicated time points after the chase were chilled on ice and, subsequently, cells were separated from the growth medium by centrifugation. The appearance of BPN’ in the growth medium fractions was then analyzed by immunoprecipitation with antibodies against BPN’, LDS-PAGE and autoradiography. The position of mature BPN’ (m) is indicated

The kinetics of precursor protein processing to the mature form can be used as a measure for the rate of protein secretion as signal peptide cleavage by signal peptidase is dependent on membrane translocation of the respective precursor protein [22, 35]. To analyze the effects of the different mutations in secretion machinery components or cell envelope-associated proteases on the rates of AmyE and AmyL secretion, pulse-chase labeling experiments with [^35^S]-methionine were performed [26]. Notably, in the case of BPN’, it was impossible to detect the short-lived [^35^S]-labelled precursor forms in the cells by immunoprecipitation, because the strong proteolytic activity of BPN’ results in antibody degradation [26]. Therefore, effects of different mutations on the kinetics of BPN’ secretion were assessed by measuring the appearance of [^35^S]-labeled mature BPN’ in the growth medium. Interestingly, the only mutations that exerted major kinetic effects on the secretion of individual reporter proteins were the *secDF*, *secG* and *dnaK* mutations. In particular, the *secDF* mutation had a significant impact on the rates of AmyE and AmyL processing, but barely affected the secretion rate of BPN’ (Fig. 4). The deletion of *secG* had a major impact on the extracellular appearance of BPN’, but it did not detectably affect the processing rates of AmyE or AmyL during the time frame of the pulse-chase labeling experiment. Interestingly, the secretion rate of BPN’ was most severely affected by the *dnaK* mutation. None of the other investigated mutations showed strong detectable kinetic effects on the secretion of AmyE, AmyL or BPN’.

### Cellular levels of HtrA and HtrB as read-out for secretion and cell envelope stress responses

**Fig. 5:**
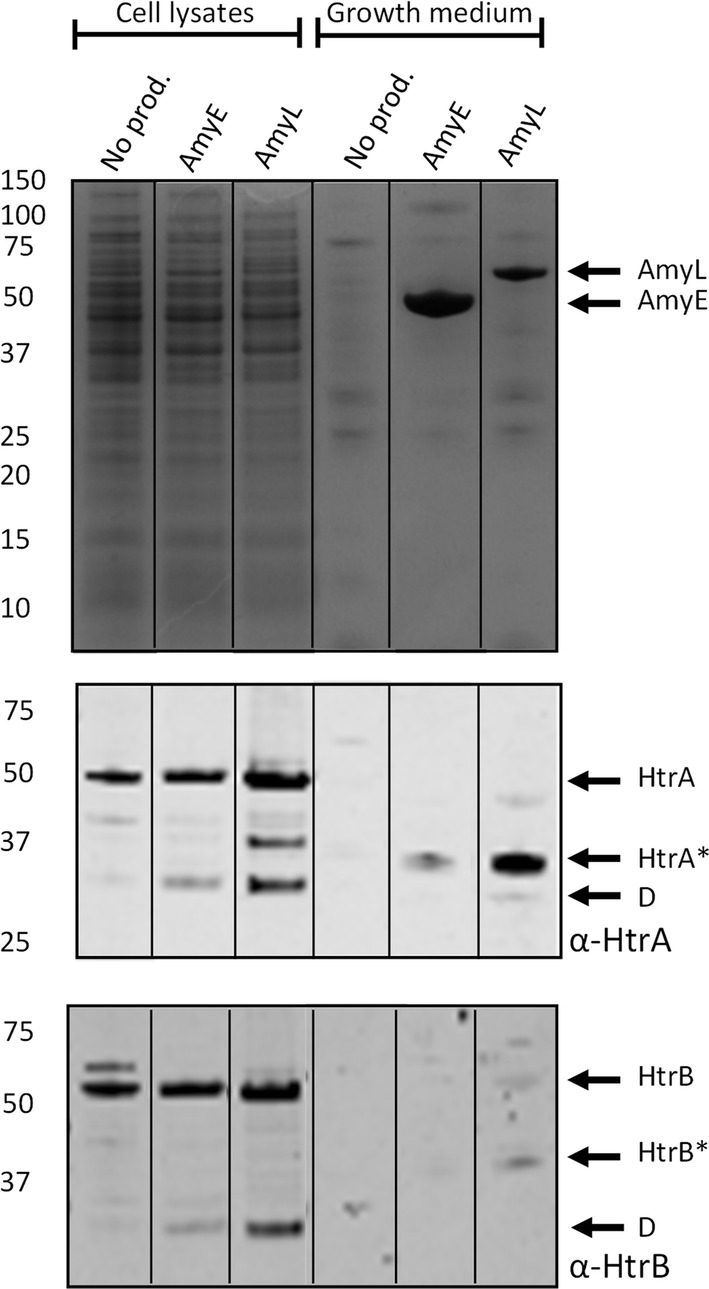
Expression of HtrA and HtrB upon AmyE or AmyL production. Wild-type cells producing AmyE or AmyL were separated from the growth medium by centrifugation after 16 h of growth in MBU medium at 37 ℃. Subsequently, proteins in the cells and growth medium fractions were separated by LDS-PAGE, and visualized with SimplyBlue SafeStain as described for Figure 1 (upper panel). The presence of HtrA and HtrB in the cell and growth medium fractions was analyzed by Western blotting using polyclonal antibodies against HtrA (middle panel) or HtrB (lower panel). The extracellular proteolytically processed forms of HtrA and HtrB are marked with a star. Major cell-associated degradation products are marked with a ‘D’. Molecular weights of marker proteins are indicated (in kDa) on the left side of each gel and Western blot

The high-level production of secretory proteins in *B. subtilis* is known to be stressful for the bacterial cells [28, 44]. Accordingly, they mount several responses to counteract this stress, in particular the CssRS-dependent secretion stress response [28, 33, 45–47]. While the impact of secretory protein production on this secretion stress induction has been investigated quite extensively, the possible impact of mutations in the secretion machinery on secretion stress was thus far ignored. To gain a better understanding of the interplay between the secretion machinery, cell envelope-associated proteases and the CssRS-dependent stress response, we decided to assess secretion stress induction by measuring the cellular levels of the major CssRS-controlled proteins HtrA and HtrB by Western blotting. Of note, HtrA and HtrB induction can also be detected in the growth medium (Fig. 5) but, as previously shown, the extracellular levels of their proteolytically processed forms depend critically on the levels of RasP and the eight secreted proteases of *B. subtilis*, especially WprA [30, 34, 37, 39, 48]. Hence, the cellular HtrA and HtrB levels are reflecting secretion stress induction more reliably than the extracellular levels and, importantly, they directly reflect the levels of the main effectors regulated by the secretion stress response.

As shown in Fig. 5, the cellular levels of HtrA and HtrB are significantly induced upon production of AmyL, which is consistent with previous findings showing secretion stress induction by the production of AmyQ or AmyM from *Geobacillus stearothermophilus* [49]. In contrast, AmyE production resulted in a relatively moderate induction of HtrA and HtrB, despite the fact that AmyE was produced at a much higher level than AmyL (Figs. 1 and 5). Conceivably, this relates to the fact that the native AmyE protein has co-evolved with *B. subtilis*, whereas AmyL, AmyQ and AmyM are derived from other *Bacillus* species.

### Impact of non-essential secretion machinery mutations on the secretion stress response

**Fig. 6:**
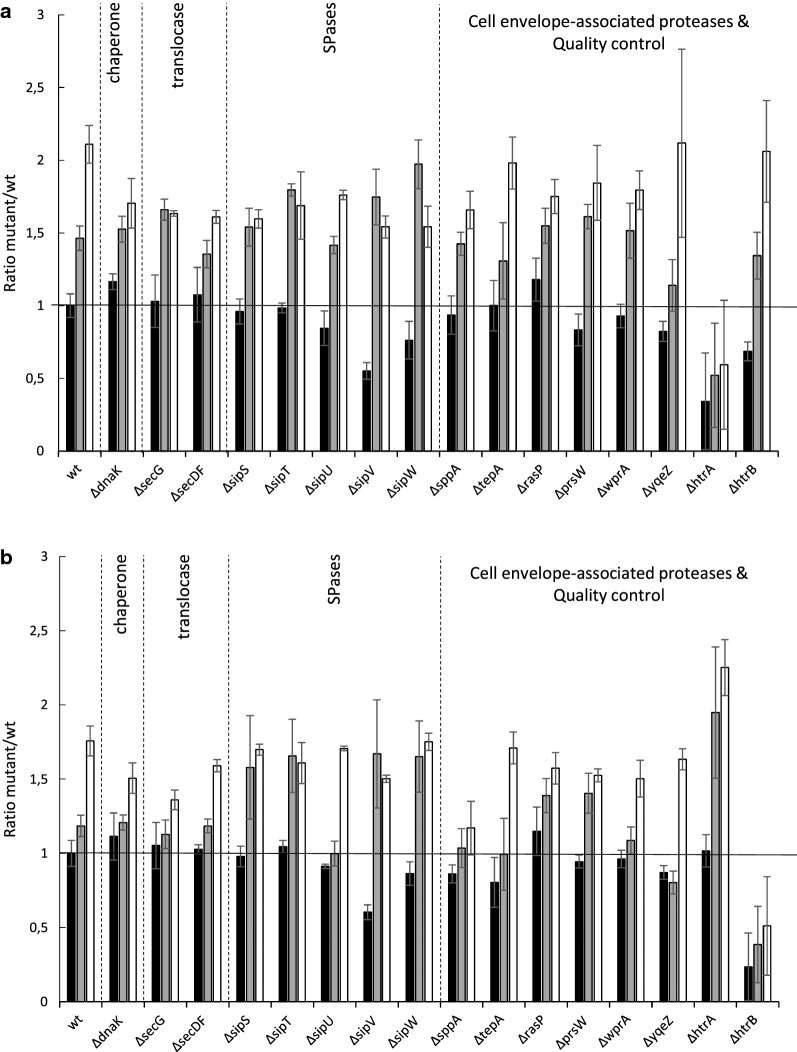
Analysis of HtrA and HtrB levels in strains lacking individual non-essential secretion machinery components or cell envelope-associated proteases upon production of AmyE or AmyL. The levels of full-size HtrA (**a**) or HtrB (**b**) in wild-type or mutant cells producing AmyE or AmyL was assessed by Western blotting with specific antibodies as described for Fig. 5. The relative levels of HtrA or HtrB compared to the respective levels in the wild-type strain were assessed by ImageJ analysis. Black bars represent the HtrA or HtrB levels in non-producing strains, grey bars relate to the HtrA or HtrB levels in AmyE-producing strains, and white bars to HtrA or HtrB levels in AmyL-producing strains. The error bars represent the standard error of the mean for three independent experiments

Following the establishment of base-line secretion stress levels in our reporter strains, we assessed the cellular HtrA and HtrB levels in the different mutant strains lacking non-essential secretion machinery components or cell envelope-associated proteases as shown in Fig. 6. To this end, the AmyE- or AmyL-producing strains, or the corresponding non-producing mutant strains were grown for 16 to 17 h in MBU medium and the HtrA and HtrB levels were assessed by Western blotting. To focus on the intact effector proteins and to ensure comparability of the data, only the full-size forms of the cellular HtrA and HtrB proteins were quantified. Of note, BPN’-producing strains were excluded from this analysis, because this serine protease degrades the cell-associated HtrA and HtrB proteins (not shown).

When the HtrA and HtrB levels were compared in non-producing strains, relatively little variation was observed with exception of the *sipV* mutant (Fig. 6). In this mutant the cellular HtrA and HtrB levels drop to almost 50% of the respective wild-type levels. Another noteworthy finding was that, in contrast to previous studies [33, 37, 38], no cross-regulation of *htrA* and *htrB* was detectable in the non-producing cells under the applied conditions. In fact, the HtrA level was even reduced in the *htrB* mutant cells (Fig. 6a).

In contrast to the non-producing cells, some differences in HtrA or HtrB production were observed in amylase-producing mutants that lack particular secretion machinery components or cell envelope-associated proteases. As for AmyE-producing cells, elevated HtrA levels were observed for *sipT*, *sipV*, and *sipW* mutant cells, while elevated HtrB levels were observed in *sipT*, *sipV*, *sipW*, and *htrA* mutant cells (Fig. 6). The strong induction of HtrA and HtrB in *sipV* mutant cells that produce AmyE as compared to non-producing cells is particularly noteworthy. Further, it is noteworthy that the HtrB level is increased in AmyE-producing cells lacking *htrA*, consistent with the previously reported cross-regulation of *htrA* and *htrB*.

Lastly, as shown in Fig. 6, the effect of AmyL production on the cellular HtrA and HtrB levels was quite different from that of AmyE production. Essentially, the HtrA levels in all mutant cells producing AmyL were slightly lower, or at best equal to the levels in the parental cells. A similar trend was observed for the HtrB levels, where the strongest reduction was observed for the *sppA* mutant producing AmyL.

## Discussion

In the present study, we investigated the contributions of non-essential Sec-pathway components and cell envelope-associated proteases on secretion of the α-amylases AmyE and AmyL, and the serine protease BPN’. Our present observations show that, of all the non-essential factors previously implicated in secretory protein production, SecDF, SecG and RasP have the strongest impact on high-level secretion of AmyE, AmyL and BPN’.

A clear advantage of our present experimental setup is that we introduced all mutations for Sec pathway components and cell envelope-associated proteases in the same genetic background and assayed their effects on protein secretion under the same growth conditions. A possible limitation of our experimental setup is that we used bacterial cultures in shake flasks, which is less optimal than the use of bioreactors. However, given the number of mutations investigated in combination with the overproduction of three different reporter proteins, it was logistically not feasible for us to perform the present comparative analyses in bioreactors. Hence, the best possible alternative option was to perform the cultivations in shake flasks under production-mimicking conditions, where the cultures reached OD_600_ values of close to 30. Although the growth curves of the various wild-type and mutant strains were comparable, with or without overexpression of secretory proteins, they were not identical. This is reflected in the optical densities of the cultures at different time points, as exemplified in Fig. 1. Such inevitable differences in growth may have influenced to some extent the protein production levels.

Another advantage of the present experimental setup was that the export kinetics of the three secretory target proteins by the mutant strains could be investigated by pulse-chase labeling. In this respect, it should be noted that the time frame of our pulse-chase labeling experiments (90 s for AmyE and AmyL, and up to 30 min for BPN’; Fig. 4) is short compared to the 16 to 17 h of culturing in the experiments where the yields for AmyE, AmyL or BPN’ were assessed by LDS-PAGE and SimplyBlue staining. Thus, it is well conceivable that small differences in the secretion kinetics (e.g. of AmyE and AmyL in the *secG* mutant, or BPN’ in the *secDF* mutant) are not clearly detectable upon pulse-chase labeling, but still do impact on the secretory protein yields after 16 to 17 h of culturing. Further, the pulse-chase labeling experiments revealed remarkable secretion kinetics of BPN’, showing that processing of its pro-peptide and secretion into the medium are very fast in a wild-type background where, essentially, everything happens within the 30 s of labeling with [^35^S]-methionine. Clear secretion kinetics for BPN’ could only be observed in the *dnaK* mutant, similar to what we have previously shown for the *rasP* mutant [26]. Importantly, the combined results from the shake flask and pulse-chase labeling experiments allowed us to narrow down the key non-essential determinants for protein secretion in *B. subtilis* to SecDF, SecG and RasP.

Of note, our results show that the precise impact of *secDF*, *secG* and *rasP* mutations depends on the investigated secretory protein. Since SecG is a component of the membrane-embedded SecYEG translocation channel, the differential impact of this protein on the secretion of AmyE, AmyL and BPN’ is likely due to differences in structural or conformational features of the translocated reporter proteins. Likewise, differential effects of the absence of SecDF may relate to differences in the proton-motive force dependency or post-translocational folding of different secretory proteins, since SecDF is a proton-driven motor for protein export implicated in late stages of translocation [19, 50]. Differential effects of the *rasP* mutation are suggestive of differences in the clearance of mis-localized precursor proteins, especially since the same signal peptide was used to secrete AmyE, AmyL and BPN’. Remarkably, we observed that the chaperone DnaK is important only for optimal secretion of BPN’, but not for AmyE or AmyL secretion. This could indicate that BPN’ may have different requirements for preventing its folding in the cytoplasm prior to membrane translocation than AmyE and AmyL [51]. However, since DnaK is a general chaperone, the observed effect of the *dnaK* deletion on BPN’ secretion could also be indirectly exerted via, as yet unidentified, cellular components that require DnaK for proper functioning.

The present findings are complementary to overexpression approaches where individual secretion machinery components were overexpressed. In particular, we have previously shown that overexpression of RasP resolves important secretion bottlenecks for difficult-to-produce enzymes, such as a serine protease from *Bacillus clausii* and the α-amylase AmyAc from *Paenibacillus curdlanolyticus* [26]. Likewise, Chen et al. [52] showed that the overexpression of *secDF* led to enhanced secretion of AmyL and the α-amylase AmyS from *Geobacillus stearothermophilus*. The latter is consistent with previous and present observations that SecDF is of major importance for protein secretion in *B. subtilis* [19]. Nevertheless, overexpression of *secG* did not result in improved secretion efficiencies [20, 52]. On the other hand, we observed in the present study that the deletion of certain genes, like the *sip* genes, had more limited effects on secretion of AmyE, AmyL and BPN’, whereas previous studies showed that their overproduction can lead to improved secretion of particular reporter proteins [53–55]. However, in case of the signal peptidases, the limited effects of single *sip* gene deletions can be attributed to the functional redundancy of the five paralogous enzymes, whereas differential effects upon overproduction can be related to their different substrate preferences [21, 23]. In fact, the differential substrate preferences of the *B. subtilis* signal peptidases are most likely the reason why deletion of particular *sip* genes may result in improved production of particular secretory proteins [21]. In this context, it should again be noted that the three reporter proteins used in the present study were all targeted for secretion with the same signal peptide. This implies that the mature proteins do impact to some extent on signal peptide processing by signal peptidase. Consistent with this observation, it has been reported that, apart from the signal peptide, also so-called ‘multiple targeting signals’ are located within the mature parts of secretory precursor proteins, which are important for translocation [56].

Different substrate preferences of the five *B. subtilis* signal peptidases may also explain why *sip* mutations showed the highest differential impact on the cellular levels of HtrA and HtrB. Especially, in the absence of SipV, the cellular HtrA and HtrB levels were signficantly decreased compared to the wild-type situation. At present, we can only speculate about the reason for this reduction. A previous study has shown that SipV is involved in the processing and secretion of the lipoteichoic acid synthase YfnI [57]. Thus, it is conceivable that in the absence of YfnI cleavage by SipV the cellular lipoteichoic acid levels increase, potentially leading to a more negatively charged cell wall. It was shown in a previous study that an increase in the negative charge of the cell wall leads to a reduced level of CssRS-dependent expression of HtrA and HtrB [51] and, accordingly, increased YfnI activity in the absence of SipV could lead to reduced levels of these secretion stress reporters. Additionally, also the levels of cellular HtrA and HtrB in the *sipT*, *sipV* and *sipW* mutants expressing AmyE were increased. It is presently difficult to reconcile the higher HtrA and HtrB levels in these mutant cells with the AmyE production levels, but some of these effects could be indirect as signal peptidases may be involved in HtrA and/or HtrB processing and secretion. Also, in the case of AmyL production, it is difficult to reconcile the observed HtrA and HtrB levels with the different investigated mutations in secretion machinery components or cell envelope-associated proteases. This is particularly surprising in case of the *secDF*, *secG* and *rasP* mutations that impact significantly on α-amylase secretion and it probably reflects the pleiotropic effects of these mutations on the native secreted proteins of *B. subtilis*. Yet, the signal peptidase redundancy is probably advantageous from an evolutionary perspective, as *Bacillus* species like *B. subtilis* evolved to secrete many different proteins with extensive variations in their signal peptides and mature protein sequences, overall sizes and pI.

## Conclusions

A likely consequence of the extensive variations in the secretory protein portfolio of *B. subtilis* is that this bacterium’s secretion machinery is ‘good enough’ for providing a competitive advantage in its ecological niche, the soil and plant rhizosphere, but not tuned for the optimal secretion of individual heterologous proteins in an industrial context. This is consistent with the view that different secretory proteins have to face different secretion bottlenecks and, accordingly, our present observations with the secretory reporter proteins AmyE, AmyL and BPN’ cannot be directly extrapolated to other recombinant secretory proteins. On the other hand, the ‘consensus nature’ of the *B. subtilis* protein secretion machinery creates opportunities for strain engineering approaches to improve secretion. For instance, an improved potential for protein secretion may be achieved by reducing the numbers of secreted proteins that compete for export with particular secretory proteins of interest through genome minimization [39], and by altering the expression of the most important secretion machinery components [26, 52].

## Materials and methods

### Bacterial strains and growth conditions

Strains and plasmids used in this study are listed in Additional file 1: Table S1. *B. subtilis* strains were grown at 37 ℃, under vigorous shaking (280 rpm) in Lysogeny Broth (LB; Oxoid Limited) or MBU medium [26]. If appropriate, the media were supplemented with chloramphenicol (2.5 µg/ml), neomycin (15 µg/ml), phleomycin (4 µg/ml) or spectinomycin (100 µg/ml). To select for amplified amylase or protease reporter genes, chloramphenicol was used at 25 µg/ml as described [26].

### Strain construction

Ex-Taq polymerase, dNTPs and buffers used for the construction of the mutant strains were purchased from Takara Bio Inc. (Shiga, Japan). Primers were obtained from Eurogentec (Maastricht, The Netherlands). Construction of deletion mutants in *B. subtilis* was performed using the modified mutation delivery method in the strain CB-15-14Δ*upp* as described by Fabret et al. [58]. To completely replace the target gene by a phleomycin resistance cassette fused to *upp* and *cI*, 5′ and 3′ flanking regions of these genes were amplified using primer combinations designated P1/P2 and P3/P4 for each respective target (Additional file 1: Table S2). The resulting PCR fusion product was used to transform cells of the *B. subtilis* Δ*upp::neoR* strain, where expression of the competence transcription factor ComK was induced with 0.3% xylose. Correct removal of the gene of interest was confirmed by PCR using primer combinations P0/P4 and P0/CI2.rev. Overproduction of AmyE [59], AmyL [60] or BPN’-Y217L (in short BPN’) [61, 62] using the *aprE* promoter and signal sequence was achieved as previously described [26].

### Analysis of secreted protein production by LDS-PAGE and Western blotting

Cultures were inoculated from LB plates with 25 μg/ml chloramphenicol and grown for approximately 8 h in LB broth with 25 μg/ml chloramphenicol. These cultures were diluted 1000-fold in MBU medium with 2.5 μg/ml chloramphenicol in Ultra Yield Flasks™ (Thomson Instrument Company) and incubated for approximately 16 h at 37 °C, 280 rpm in a Multitron orbital shaker (Infors) at high humidity. After measuring and correcting for the OD_600_, equal amounts of cells were separated from the culture medium by centrifugation. For the analysis of extracellular proteins, proteins in the culture medium were precipitated with trichloroacetic acid (TCA; 10% w/v final concentration), dissolved in LDS buffer (Life Technologies) and heated for 10 min at 95 °C. To assess cellular proteins, the cell pellets were resuspended in 0.2 M HCl to inhibit protease activity and disrupted by bead-beating with 0.1 µm glass beads (Biospec Products, Bartlesville, USA) using a Precellys24 bead beater (Bertin Technologies, Montigny-le-Bretonneux, France). The resulting lysates were incubated for 10 min at 0 °C. Samples of cellular and extracellular proteins were mixed with LDS gel loading buffer (Life Technologies), and the proteins were subsequently separated by LDS-PAGE on 10% NuPage gels (Life Technologies). Gels were stained with SimplyBlue™ SafeStain (Life Technologies). Each experiment was performed at least three times.

For Western blotting, proteins were transferred to a nitrocellulose membrane (Protran^®^, Schleicher & Schuell, Dassel, Germany). Immunodetection was performed using rabbit polyclonal antibodies raised against TrxA, HtrA or HtrB (Eurogentec). Visualization of bound primary antibodies was performed by using fluorescently labeled secondary antibodies (IRDye 800 CW from LiCor Biosciences, Nebraska, USA). Membranes were scanned for fluorescence at 800 nm using the Odyssey Infrared Imaging System (LiCor Biosciences) and images were quantified with the ImageJ software package (http://imagej.nih.gov/ij/). Each experiment was performed at least two or three times.

### Pulse-chase protein labeling experiments

Pulse-chase labeling of *B. subtilis* proteins was performed using Easy tag [^35^S]-methionine (PerkinElmer Inc.) followed by immunoprecipitation and LDS-PAGE as described previously [26, 63]. Cells were grown for 16 h in MBU supplemented with chloramphenicol and diluted 1 h before the actual labeling to OD_600_ ~ 0.7 in fresh MBU with chloramphenicol. Labeling was performed with 25 µCi [^35^S]-methionine for 30 s before adding an excess amount of unlabeled methionine (chase; 0.625 mg/ml final concentration). Samples were collected at several time points, followed by direct precipitation of the proteins with 10% TCA on ice. Precipitates were resuspended in lysis buffer (10 mM Tris pH 8, 25 mM MgCl2, 200 mM NaCl and 5 mg/ml lysozyme). After 10–15 min incubation at 37 °C, lysis was achieved by adding 1% (w/v) SDS and heating for 10 min at 100 °C. Specific rabbit polyclonal antibodies against AmyE or AmyL were used for immunoprecipitation of the respective labeled proteins in STD-Tris buffer (10 mM Tris pH 8.2, 0.9% (w/v) NaCl, 1.0% (v/v)Triton X-100, 0.5% (w/v) sodium deoxycholate) with the help of Protein A affinity medium (Mabselect Sule, GE Healthcare Life Sciences). Because of the high proteolytic activity of BPN’, which also degrades antibodies, the immunoprecipitation of BPN’ with rabbit polyclonal antibodies was performed in the presence of the Pefablock SC serine protease inhibitor (4 mM; Roche). Labelled proteins were separated by LDS-PAGE using 10% NuPage gels (Life Technologies) and visualized using a Cyclon Plus Phosphor Imager (Perkin Elmer). Each experiment was performed at least two times.

## Supplementary information

**Additional file 1: Table S1.** Strains used in this study. **Table S2.** Primers used in this study.

## Data Availability

All data generated or analyzed during this study are included in this published article and its supplementary information files.
