# Supplementary material for: Relative contributions of non-essential Sec pathway components and cell envelope-associated proteases to high-level enzyme secretion by Bacillus subtilis
Source: Microb Cell Fact. 2020 Feb 28;19:52. doi: 10.1186/s12934-020-01315-2 (PMC7048088; doi:10.1186/s12934-020-01315-2)
Supplement: Supplementary file 1 — Additional file 1: Table S1. Strains used in this study. Table S2. Primers used in this study. [file 12934_2020_1315_MOESM1_ESM.pdf]

Additional file 1 to:

Relative contributions of non-essential Sec pathway components and cell envelope-associated proteases to high-level enzyme secretion by *Bacillus subtilis*

Jolanda Neef[1], Cristina Bongiorni[2], Brian Schmidt[2], Vivianne J. Goosens[1], Jan Maarten van Dijl[1#].

[1]University of Groningen, University Medical Center Groningen, Department of Medical Microbiology, Hanzeplein 1, P.O. Box 30001, 9700 RB Groningen, The Netherlands

[2]DuPont Industrial Biosciences, 925 Page Mill Road, Palo Alto, CA 94304, United States

Table S1: Strains used in this study

B. subtilis strain	Relevant genotypes and phenotypes	Source or reference
Δ*upp*	*degUHy32*, *amyE*::*xylRPxylAcomK-ermC*, Δ*upp::neoR*; Em^{R} , Neo^{R}	[1]
AmyE	*degUHy32, amyE::xylRPxylAcomK-ermC, aprE::PaprE-amyE catR* Tbpn'; Em^{R} ; Cm^{R}	[1]
AmyL	*degUHy32, amyE::xylRPxylAcomK-ermC, aprE::PaprE-amyL catR* Tbpn'; Em^{R} ; Cm^{R}	[1]
BPN'	*degUHy*32, *amyE::xylRPxylAcomK-ermC, aprE::PaprE-bpn' catR* Tbpn'; Em^{R} ; Cm^{R}	[1]
Δ*dnaK*	*degUHy32, amyE::xylRPxylAcomK-ermC, Δupp::neoR, ΔdnaK::upp-phleoR-cI* Em^{R}; Phleo^{R}; Neo^{S}	This study
Δ*secG*	*degUHy32, amyE::xylRPxylAcomK-ermC, Δupp::neoR, ΔsecG::upp-phleoR-cI* Em^{R}; Phleo^{R}; Neo^{S}	This study
Δ*secDF*	*degUHy32, amyE::xylRPxylAcomK-ermC, Δupp::neoR, ΔsecDF::upp-phleoR-cI* Em^{R}; Phleo^{R}; Neo^{S}	This study
Δ*sipS*	*degUHy32, amyE::xylRPxylAcomK-ermC, Δupp::neoR, ΔsipS::upp-phleoR-cI* Em^{R}; Phleo^{R}; Neo^{S}	This study
Δ*sipT*	*degUHy32, amyE::xylRPxylAcomK-ermC, Δupp::neoR, ΔsipT::upp-phleoR-cI* Em^{R}; Phleo^{R}; Neo^{S}	This study
Δ*sipU*	*degUHy32, amyE::xylRPxylAcomK-ermC, Δupp::neoR, ΔsipU::upp-phleoR-cI* Em^{R}; Phleo^{R}; Neo^{S}	This study
Δ*sipV*	*degUHy32, amyE::xylRPxylAcomK-ermC, Δupp::neoR, ΔsipV::upp-phleoR-cI* Em^{R}; Phleo^{R}; Neo^{S}	This study
Δ*sipW*	*degUHy32, amyE::xylRPxylAcomK-ermC, Δupp::neoR, ΔsipW::upp-phleoR-cI* Em^{R}; Phleo^{R}; Neo^{S}	This study
Δ*sppA*	*degUHy32, amyE::xylRPxylAcomK-ermC, Δupp::neoR, ΔsppA::upp-phleoR-cI* Em^{R}; Phleo^{R}; Neo^{S}	This study
Δ*tepA*	*degUHy32, amyE::xylRPxylAcomK-ermC, Δupp::neoR, ΔtepA::upp-phleoR-cI* Em^{R}; Phleo^{R}; Neo^{S}	This study
Δ*prsW*	*degUHy32, amyE::xylRPxylAcomK-ermC, Δupp::neoR, ΔprsW::upp-phleoR-cI* Em^{R}; Phleo^{R}; Neo^{S}	This study
Δ*wprA*	*degUHy32, amyE::xylRPxylAcomK-ermC, Δupp::neoR, ΔwprA::specR* Em^{R}; Spec^{R}; Neo^{R}	This study
Δ*htrA*	*degUHy32, amyE::xylRPxylAcomK-ermC, Δupp::neoR, ΔhtrA::upp-phleoR-cI* Em^{R}; Phleo^{R}; Neo^{S}	This study

*Δ**htrB*	*degUHy32, amyE::xylRPxylAcomK-ermC, Δupp::neoR, ΔhtrB::upp-phleoR-cI* Em^R; $Phleo^R$; Neo^S	This study
ΔyqeZ	*degUHy32, amyE::xylRPxylAcomK-ermC, Δupp::neoR, ΔyqeZ::upp-phleoR-cI* Em^R; $Phleo^R$; Neo^S	This study
AmyE Δ*dnaK*	*degUHy32, amyE::xylRPxylAcomK-ermC, Δupp::neoR, ΔdnaK::upp-phleoR-cI aprE::PaprE-amyE catR* T*bpn'* Em^R; $Phleo^R$; Neo^S; Cm^R	This study
AmyE *ΔsecG*	*degUHy32, amyE::xylRPxylAcomK-ermC, Δupp::neoR, ΔsecG::upp-phleoR-cI aprE::PaprE-amyE catR* T*bpn'* Em^R; $Phleo^R$; Neo^S; Cm^R	This study
AmyE *ΔsecDF*	*degUHy32, amyE::xylRPxylAcomK-ermC, Δupp::neoR, ΔsecDF::upp-phleoR-cI aprE::PaprE-amyE catR* T*bpn'* Em^R; $Phleo^R$; Neo^S; Cm^R	This study
AmyE *ΔsipS*	*degUHy32, amyE::xylRPxylAcomK-ermC, Δupp::neoR, ΔsipS::upp-phleoR-cI aprE::PaprE-amyE catR* T*bpn'* Em^R; $Phleo^R$; Neo^S; Cm^R	This study
AmyE *ΔsipT*	*degUHy32, amyE::xylRPxylAcomK-ermC, Δupp::neoR, ΔsipT::upp-phleoR-cI aprE::PaprE-amyE catR* T*bpn'* Em^R; $Phleo^R$; Neo^S; Cm^R	This study
AmyE *ΔsipU*	*degUHy32, amyE::xylRPxylAcomK-ermC, Δupp::neoR, ΔsipU::upp-phleoR-cI aprE::PaprE-amyE catR* T*bpn'* Em^R; $Phleo^R$; Neo^S; Cm^R	This study
AmyE *ΔsipV*	*degUHy32, amyE::xylRPxylAcomK-ermC, Δupp::neoR, ΔsipV::upp-phleoR-cI aprE::PaprE-amyE catR* T*bpn'* Em^R; $Phleo^R$; Neo^S; Cm^R	This study
AmyE *ΔsipW*	*degUHy32, amyE::xylRPxylAcomK-ermC, Δupp::neoR, ΔsipW::upp-phleoR-cI aprE::PaprE-amyE catR* T*bpn'* Em^R; $Phleo^R$; Neo^S; Cm^R	This study
AmyE *ΔsppA*	*degUHy32, amyE::xylRPxylAcomK-ermC, Δupp::neoR, ΔsppA::upp-phleoR-cI aprE::PaprE-amyE catR* T*bpn'* Em^R; $Phleo^R$; Neo^S; Cm^R	This study

AmyE *ΔtepA*	*degUHy32, amyE::xylRPxylAcomK-ermC, Δupp::neoR, ΔtepA::upp-phleoR-cI aprE::PaprE-amyE catR* T*bpn’* Em^R; $Phleo^R$; Neo^S; Cm^R	This study
AmyE *ΔprsW*	*degUHy32, amyE::xylRPxylAcomK-ermC, Δupp::neoR, ΔprsW::upp-phleoR-cI aprE::PaprE-amyE catR* T*bpn’* Em^R; $Phleo^R$; Neo^S; Cm^R	This study
AmyE *ΔwprA*	*degUHy32, amyE::xylRPxylAcomK-ermC, Δupp::neoR, ΔwprA::specR aprE::PaprE-amyE catR* T*bpn’* Em^R; $Spec^R$; Neo^R; Cm^R	This study
AmyE *ΔhtrA*	*degUHy32, amyE::xylRPxylAcomK-ermC, Δupp::neoR, ΔhtrA::upp-phleoR-cI aprE::PaprE-amyE catR* T*bpn’* Em^R; $Phleo^R$; Neo^S; Cm^R	This study
AmyE *ΔhtrB*	*degUHy32, amyE::xylRPxylAcomK-ermC, Δupp::neoR, ΔhtrB::upp-phleoR-cI aprE::PaprE-amyE catR* T*bpn’* Em^R; $Phleo^R$; Neo^S; Cm^R	This study
AmyE *ΔyqeZ*	*degUHy32, amyE::xylRPxylAcomK-ermC, Δupp::neoR, ΔyqeZ::upp-phleoR-cI aprE::PaprE-amyE catR* T*bpn’* Em^R; $Phleo^R$; Neo^S; Cm^R	This study
AmyL Δ*dnaK*	*degUHy32, amyE::xylRPxylAcomK-ermC, Δupp::neoR, ΔdnaK::upp-phleoR-cI aprE::PaprE-amyL catR* T*bpn’* Em^R; $Phleo^R$; Neo^S; Cm^R	This study
AmyL *ΔsecG*	*degUHy32, amyE::xylRPxylAcomK-ermC, Δupp::neoR, ΔsecG::upp-phleoR-cI aprE::PaprE-amyL catR* T*bpn’* Em^R; $Phleo^R$; Neo^S; Cm^R	This study
AmyL *ΔsecDF*	*degUHy32, amyE::xylRPxylAcomK-ermC, Δupp::neoR, ΔsecDF::upp-phleoR-cI aprE::PaprE-amyL catR* T*bpn’* Em^R; $Phleo^R$; Neo^S; Cm^R	This study
AmyL *ΔsipS*	*degUHy32, amyE::xylRPxylAcomK-ermC, Δupp::neoR, ΔsipS::upp-phleoR-cI aprE::PaprE-amyL catR* T*bpn’* Em^R; $Phleo^R$; Neo^S; Cm^R	This study

AmyL Δ*sipT*	*degUHy32, amyE::xylRPxylAcomK-ermC, Δupp::neoR, ΔsipT::upp-phleoR-cI aprE::PaprE-amyL catR* T*bpn'* Em^R; $Phleo^R$; Neo^S; Cm^R	This study
AmyL Δ*sipU*	*degUHy32, amyE::xylRPxylAcomK-ermC, Δupp::neoR, ΔsipU::upp-phleoR-cI aprE::PaprE-amyL catR* T*bpn'* Em^R; $Phleo^R$; Neo^S; Cm^R	This study
AmyL Δ*sipV*	*degUHy32, amyE::xylRPxylAcomK-ermC, Δupp::neoR, ΔsipV::upp-phleoR-cI aprE::PaprE-amyL catR* T*bpn'* Em^R; $Phleo^R$; Neo^S; Cm^R	This study
AmyL Δ*sipW*	*degUHy32, amyE::xylRPxylAcomK-ermC, Δupp::neoR, ΔsipW::upp-phleoR-cI aprE::PaprE-amyL catR* T*bpn'* Em^R; $Phleo^R$; Neo^S; Cm^R	This study
AmyL Δ*sppA*	*degUHy32, amyE::xylRPxylAcomK-ermC, Δupp::neoR, ΔsppA::upp-phleoR-cI aprE::PaprE-amyL catR* T*bpn'* Em^R; $Phleo^R$; Neo^S; Cm^R	This study
AmyL Δ*tepA*	*degUHy32, amyE::xylRPxylAcomK-ermC, Δupp::neoR, ΔtepA::upp-phleoR-cI aprE::PaprE-amyL catR* T*bpn'* Em^R; $Phleo^R$; Neo^S; Cm^R	This study
AmyL Δ*prsW*	*degUHy32, amyE::xylRPxylAcomK-ermC, Δupp::neoR, ΔprsW::upp-phleoR-cI aprE::PaprE-amyL catR* T*bpn'* Em^R; $Phleo^R$; Neo^S; Cm^R	This study
AmyL Δ*wprA*	*degUHy32, amyE::xylRPxylAcomK-ermC, Δupp::neoR, ΔwprA::specR aprE::PaprE-amyL catR* T*bpn'* Em^R; $Spec^R$; Neo^R; Cm^R	This study
AmyL Δ*htrA*	*degUHy32, amyE::xylRPxylAcomK-ermC, Δupp::neoR, ΔhtrA::upp-phleoR-cI aprE::PaprE-amyL catR* T*bpn'* Em^R; $Phleo^R$; Neo^S; Cm^R	This study
AmyL Δ*htrB*	*degUHy32, amyE::xylRPxylAcomK-ermC, Δupp::neoR, ΔhtrB::upp-phleoR-cI aprE::PaprE-amyL catR* T*bpn'* Em^R; $Phleo^R$; Neo^S; Cm^R	This study

AmyL *ΔyqeZ*	*degUHy32, amyE::xylRPxylAcomK-ermC, Δupp::neoR, ΔyqeZ::upp-phleoR-cI aprE::PaprE-amyL catR* T*bpn’* Em^{R}; Phleo^{R}; Neo^{S}; Cm^{R}	This study
BPN’ Δ*dnaK*	*degUHy32, amyE::xylRPxylAcomK-ermC, Δupp::neoR, ΔdnaK::upp-phleoR-cI aprE::PaprE-bpn’ catR* T*bpn’* Em^{R}; Phleo^{R}; Neo^{S}; Cm^{R}	This study
BPN’ *ΔsecG*	*degUHy32, amyE::xylRPxylAcomK-ermC, Δupp::neoR, ΔsecG::upp-phleoR-cI aprE::PaprE-bpn’ catR* T*bpn’* Em^{R}; Phleo^{R}; Neo^{S}; Cm^{R}	This study
BPN’ *ΔsecDF*	*degUHy32, amyE::xylRPxylAcomK-ermC, Δupp::neoR, ΔsecDF::upp-phleoR-cI aprE::PaprE-bpn’ catR* T*bpn’* Em^{R}; Phleo^{R}; Neo^{S}; Cm^{R}	This study
BPN’ *ΔsipS*	*degUHy32, amyE::xylRPxylAcomK-ermC, Δupp::neoR, ΔsipS::upp-phleoR-cI aprE::PaprE-bpn’ catR* T*bpn’* Em^{R}; Phleo^{R}; Neo^{S}; Cm^{R}	This study
BPN’ *ΔsipT*	*degUHy32, amyE::xylRPxylAcomK-ermC, Δupp::neoR, ΔsipT::upp-phleoR-cI aprE::PaprE-bpn’ catR* T*bpn’* Em^{R}; Phleo^{R}; Neo^{S}; Cm^{R}	This study
BPN’ *ΔsipU*	*degUHy32, amyE::xylRPxylAcomK-ermC, Δupp::neoR, ΔsipU::upp-phleoR-cI aprE::PaprE-bpn’ catR* T*bpn’* Em^{R}; Phleo^{R}; Neo^{S}; Cm^{R}	This study
BPN’ *ΔsipV*	*degUHy32, amyE::xylRPxylAcomK-ermC, Δupp::neoR, ΔsipV::upp-phleoR-cI aprE::PaprE-bpn’ catR* T*bpn’* Em^{R}; Phleo^{R}; Neo^{S}; Cm^{R}	This study
BPN’ *ΔsipW*	*degUHy32, amyE::xylRPxylAcomK-ermC, Δupp::neoR, ΔsipW::upp-phleoR-cI aprE::PaprE-bpn’ catR* T*bpn’* Em^{R}; Phleo^{R}; Neo^{S}; Cm^{R}	This study
BPN’ *ΔsppA*	*degUHy32, amyE::xylRPxylAcomK-ermC, Δupp::neoR, ΔsppA::upp-phleoR-cI aprE::PaprE-bpn’ catR* T*bpn’* Em^{R}; Phleo^{R}; Neo^{S}; Cm^{R}	This study

BPN’ *ΔtepA*	*degUHy32, amyE::xylRPxylAcomK-ermC, Δupp::neoR, ΔtepA::upp-phleoR-cI aprE::PaprE-bpn’ catR* T*bpn’* Em^R; $Phleo^R$; Neo^S; Cm^R	This study
BPN’ *ΔprsW*	*degUHy32, amyE::xylRPxylAcomK-ermC, Δupp::neoR, ΔprsW::upp-phleoR-cI aprE::PaprE-bpn’ catR* T*bpn’* Em^R; $Phleo^R$; Neo^S; Cm^R	This study
BPN’ *ΔwprA*	*degUHy32, amyE::xylRPxylAcomK-ermC, Δupp::neoR, ΔwprA::specR aprE::PaprE-bpn’ catR* T*bpn’* Em^R; $Spec^R$; Neo^R; Cm^R	This study
BPN’ *ΔhtrA*	*degUHy32, amyE::xylRPxylAcomK-ermC, Δupp::neoR, ΔhtrA::upp-phleoR-cI aprE::PaprE-bpn’ catR* T*bpn’* Em^R; $Phleo^R$; Neo^S; Cm^R	This study
BPN’ *ΔhtrB*	*degUHy32, amyE::xylRPxylAcomK-ermC, Δupp::neoR, ΔhtrB::upp-phleoR-cI aprE::PaprE-bpn’ catR* T*bpn’* Em^R; $Phleo^R$; Neo^S; Cm^R	This study
BPN’ *ΔyqeZ*	*degUHy32, amyE::xylRPxylAcomK-ermC, Δupp::neoR, ΔyqeZ::upp-phleoR-cI aprE::PaprE-bpn’ catR* T*bpn’* Em^R; $Phleo^R$; Neo^S; Cm^R	This study

T*bpn*’, terminator structure of *B. amyloliquefaciens bpn*’; Em^R, Erythromycin resistant; $Phleo^R$, Phleomycin resistant Neo^R, Neomycin resistant; Neo^S, Neomycin sensitive; Cm^R, Chloramphenicol resistant; $Spec^R$, Spectinomycin resistant

Table S2: Primers used in this study

Target gene	Primer name	5'→3' nucleotide sequence
	CI2.rev	CTTCAACGCTAACTTTGAG
**Chaperone**		
dnaK	dnaK.P0	CATTCATCCTCAGGACGTGTTC
	dnaK.P1	CTATGTTGACCATTTGCTGTCACC
	dnaK.P2	CGACCTGCAGGCATGCAAGCTTTGAATAACCTCCTGCTATGTAATTATTG
	dnaK.P3	CGAGCTCGAATTCACTGGCCGTCGCAATAATTACATAGCAGGAGGTTATTCAAGTTCTTTTTAGTGTCAGC CCCG
	dnaK.P4	TAAATCAAGCGGGTCCTCTATCAC
**Translocase**		
secG	secG.P0	CCAGCCTTGTACATAAGCCCC
	secG.P1	GCCTCTGCCAGTTTTTTGATG
	secG.P2	CGACCTGCAGGCATGCAAGCTCCCATACACCTCCAGACTCAC
	secG.P3	CGAGCTCGAATTCACTGGCCGTCGGTAATGTAGCCAGTGAGTCTGGAGGTGTATGGGGGCAATGTTTGTA TAAGGTCTG
	secG.P4	GAGAATGGTTTCAACCTCACCC
secDF	secDF.P0	GCACAGCTTGAAGCGGG
	secDF.P1	GGGGCCATCACTTTTATAAATACAG
	secDF.P2	CGACCTGCAGGCATGCAAGCTTATGTATATCCTCCCTTAAACCTGTGC

	secDF.P3	CGAGCTCGAATTCACTGGCCGTCGGATTCGCACAGGTTTAAGGGAGGATATACATAAAAATATCAGGCTGTCCTCTGC
	secDF.P4	CCGACAGTTTTTGCAGCG
**Signal peptidases**		
sipS	sipS.P0	CCTGACTCGGTGGCAGAAC
	sipS.P1	CCCGCCATAGACAATGTTG
	sipS.P2	CGACCTGCAGGCATGCAAGCTCTGATTTCCTCCAATAAACAACG
	sipS.P3	CGAGCTCGAATTCACTGGCCGTCGGAAGACCGTTGTTTATTGGAGGAAATCAGGATCAAGCAGCTTCCCATTG
	sipS.P4	GCAGCCTCTGACGTGATCC
sipT	sipT.P0	GGCACCAGCACCTGCC
	sipT.P1	GCCCGACAGGTATTGCC
	sipT.P2	CGACCTGCAGGCATGCAAGCTAGTGTTTCCTCCTAGACAATTGCTATG
	sipT.P3	CGAGCTCGAATTCACTGGCCGTCGCAACTGACATAGCAATTGTCTAGGAGGAAACACTAAACGCCTTGCTGGCCTAG
	sipT.P4	CAAGCACCTTTACGCCATCG
sipU	sipU.P0	GGCTTTAAGCAAACTGAACACG
	sipU.P1	GATGTGACCCGCGTAGCG
	sipU.P2	CGACCTGCAGGCATGCAAGCTCTTCCTTCACCCGATGCTTTC

	sipU.P3	CGAGCTCGAATTCACTGGCCGTCGGGAATTCTTAGGAAAGCATCGGGTGAAGGAAGAATGCAATGCAAAAAGAACATGG
	sipU.P4	CAGCTTGATATCTACATCTTCTCTTTGAAC
sipV	sipV.P0	GGGCGCGTATACCGAG
	sipV.P1	CCGAAGATGATGATGAGAGCTG
	sipV.P2	CGACCTGCAGGCATGCAAGCTGTTACTGTCTCCCTCCATCATGC
	sipV.P3	CGAGCTCGAATTCACTGGCCGTCGCAGCGTGAGCATGATGGAGGGAGACAGTAACAAAGACGCTAATTGAAGAGGCG
	sipV.P4	CCAGCAGCTCCGACATC
sipW	sipW.P0	GATGGACAGAATAAAACAGTGTAAAGG
	sipW.P1	CTCCGAGAGATGAGCGAAAAG
	sipW.P2	CGACCTGCAGGCATGCAAGCTTGCTTTTTTCATCCTCTTCCC
	sipW.P3	CGAGCTCGAATTCACTGGCCGTCGCCAGAAGGAAAGCGGGGAAGAGGATGAAAAAAGCACTTCAGTTGTAAACCTGGCA
	sipW.P4	GCCGGACTGGCTGAAATAC
**Cell envelope-associated proteases**		
sppA	sppA.P0	GCATGATCGGCTTATTCATGC
	sppA.p1	CGCATGCCGATCGCAC
	sppA.P2	CGACCTGCAGGCATGCAAGCTTTTTTCTCCTCCTTTTTCCTAAACTC

	sppA.P3	CGAGCTCGAATTCACTGGCCGTCGGAGAGAGTTTAGGAAAAAGGAGGAGAAAAAGAGGGAACAATAAT GGACGCG
	sppA.P4	CGCGATCGGCGTAATGG
tepA	tepA.P0	CGCACGGGCACGATG
	tepA.P1	CTGTCCGTTCCAGTGTACGG
	tepA.P2	CGACCTGCAGGCATGCAAGCTCTCGCTTTCATCCTTTCCG
	tepA.P3	CGAGCTCGAATTCACTGGCCGTCGCAAAGAGAAACTCGGAAAGGATGAAAGCGAGTTCTTTATACCGTGA TGCCTCAG
	tepA.P4	GGTCTGTCATTCAATTTAGACTCCAG
prsW	prsW.P0	CCAAGGCTTCTACTGGAGTGAAG
	prsW.P1	GGTAGAAGAAAAATTAGAAAAAATGATGG
	prsW.P2	CGACCTGCAGGCATGCAAGCTCAGGCAACCTCTTTCCTTTATATTC
	prsW.P3	CGAGCTCGAATTCACTGGCCGTCGGAATATAAAGGAAAGAGGTTGCCTG AAGCCGCACAGCAACCGTGC
	prsW.P4	GCGGCAGCTGACTGACGCTG
wprA	wprA.P0	GTAGAGCAAGCCCGTCCTG
	wprA.P1	GCGACAGGGGAACTTTACAAC
	wprA.P2	CGACCTGCAGGCATGCAAGCTGTTATCCCTCCTGCAAAATAATGAATC
	wprA.P3	CGAGCTCGAATTCACTGGCCGTCGCCTTCAAGGAGATTCATTATTTTGCAGGAGGGATAACCCAAAAAGC GGTGCTCG
	wprA.P4	GCCGCAATCGCTGTAATC

htrA	htrA.P0	GCCGCCCGGTCATCCCC
	htrA.P1	CCGATCGTTTATGTAAAAATGACG
	htrA.P2	CGACCTGCAGGCATGCAAGCTCATGTTCACTCCGTTTCTCTATT
	htrA.P3	CGAGCTCGAATTCACTGGCCGTCGAATAGAGAAACGGAGTGAACATGGACATAATGCCTCAGGCCG
	htrA.P4	GATACATCAAGCAGCAAGGCTTC
htrB	htrB.P0	CCGGTCCACTTTTACTGGG
	htrB.P1	CAAGCCAAATAGCCGGAATCC
	htrB.P2	CGACCTGCAGGCATGCAAGCTGTTCTTACACTCCTTTAACGGTTATTC
	htrB.P3	CGAGCTCGAATTCACTGGCCGTCGGAATAACCGTTAAAGGAGTGTAAGAACGAAAAAACAAAAAGCTGA ACCCGAT
	htrB.P4	CCATCGTGACAGGAGCGTC
yqeZ	yqeZ.P0	CGCTGCGGTCAGAAAAGG
	yqeZ.P1	CCACATGGGCATATGCTGTC
	yqeZ.P2	CGACCTGCAGGCATGCAAGCTGGGCGGTGTATCCCTCC
	yqeZ.P3	CGAGCTCGAATTCACTGGCCGTCGCTTATTATAAAAGGAAGGAGGGATACACCGCCCATAGAAACGAGGA GAAGTTATATGGATC
	yqeZ.P4	GAACGATCCCCTGTACGACG

References

1. Neef J, Bongiorni C, Goosens VJ, Schmidt B, van Dijl JM: **Intramembrane protease RasP boosts protein production in *Bacillus*.** Microb Cell Fact 2017, **16**(1):57-017.
